# Introduction to “Biomolecular Technologies”

**DOI:** 10.1039/d5cb90031j

**Published:** 2025-08-19

**Authors:** Sheel C. Dodani, Ariel Furst

**Affiliations:** a Department of Chemistry and Biochemistry, The University of Texas at Dallas Richardson Texas USA sheel.dodani@utdallas.edu; b Department of Chemical Engineering, Massachusetts Institute of Technology Cambridge MA USA afurst@mit.edu

## Abstract

As both chemical and biological engineering approaches continue to expand, the landscape of biomolecular technologies is rapidly evolving, affording new opportunities from basic science to real-world applications. This themed collection brings together engineered biomolecule-based technologies spanning small molecules, nucleic acids, and proteins, with applications in biocatalysis, biosensing, and synthetic biology. Each study showcases the modular and tunable nature of biomolecular design to tailor properties for function in both aqueous solutions and biological environments, as summarized below.

## Abstract 2

Sheel C. Dodani and Ariel Furst introduce the *RSC Chemical Biology* themed collection on Biomolecular Technologies.

## Graphical abstract

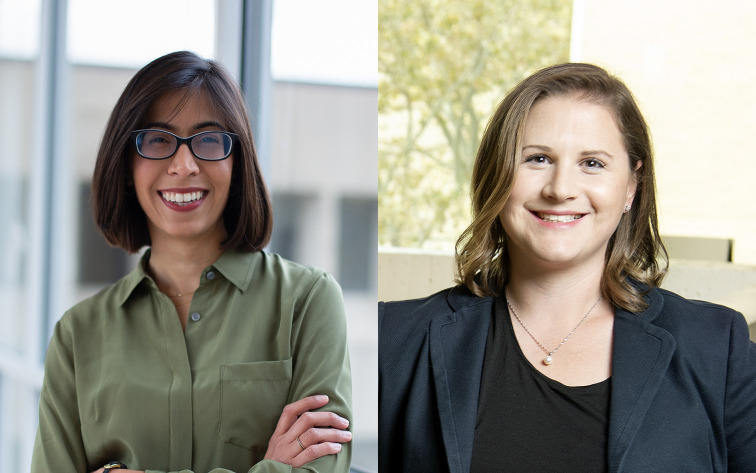

## Biocatalysis

Han and Sczepanski (https://doi.org/10.1039/D4CB00253A) describe a streamlined chemoenzymatic strategy for assembling long L-RNAs in high purity and robust yields. Using phosphoimidazolide chemistry, they generate a series of activated 5′-adenylated L-RNA donors that can be ligated to complementary L-RNA acceptors by a cross-chiral ribozyme. They extend this method to 5′-adenosyltriphosphate-activated donors to synthesize a 129-nucleotide L-RNA, the longest mirror-image RNA constructed in a single ligation step. This study addresses the need for scalable and efficient nucleic acid synthesis, advancing the reach of biocatalysis across basic research and biomanufacturing.

## Biosensing

The next three studies highlight the scope of imaging-based technologies to illuminate homeostasis and signaling in real time. Lu and colleagues (https://doi.org/10.1039/D4CB00310A) present a bioluminescent probe for imaging nitroreductase (NTR) activity as a marker of tumor progression *in vivo*. They cage the red-shifted QTZ luciferin with a nitroaromatic group (QTZ-NTR), which is selectively reduced by NTR, releasing the free substrate for NanoLuc luciferase and triggering bioluminescence. QTZ-NTR enables continuous monitoring and distinguishes NTR activity between primary and metastatic bone tumors in mouse models.

Takeuchi and colleagues (https://doi.org/10.1039/D4CB00256C) report a chemigenetic fluorescent sensor for ratiometric imaging of intracellular sodium ions (Na^+^). This work bridges synthetic small molecule design and protein engineering. In the absence of a naturally occurring Na^+^-binding protein, they convert a synthetic macrocyclic Na^+^ chelator into a HaloTag ligand to label HaloTag-GFP chimeras. Over four rounds of mutagenesis and multi-tiered screening, they develop HaloGFP-Na2.4, a sensor with physiologically relevant sodium affinity and selectivity over potassium, addressing an inherent challenge in monovalent ion detection.

Sescil and colleagues (https://doi.org/10.1039/D4CB00276H) expand the utility of the single-chain protein-based opioid transmission indicator tool (SPOTIT) for diverse biological applications. They rationally design chimeras composed of the μ-opioid G protein-coupled receptor, circularly permuted GFP, and a nanobody, connected *via* a protease-sensitive linker. This architecture enables protease-triggered opioid sensing in living cells and is further extended to monitor specific protease activity in the mouse brain. The approach yields robust signal-to-noise ratios, providing a high-contrast readout in complex biological settings.

Across all three studies, creative recognition strategies coupled with engineering, form the foundation for platform technologies, reflecting the expansive potential for biosensing across diverse targets and biological systems.

## Synthetic biology

Finally, Truong and Silberg (https://doi.org/10.1039/D4CB00257A) explore the potential of macromolecule-responsive protein switches to support electron transfer in bacteria for living electronics. For this proof-of-concept, they generate chimeras of split ferredoxin (Fd) electron carrier proteins with the GFP antigen and anti-GFP nanobodies (Nb). Among the design variables tested, both the insertion site and identity of the nanobody strongly influence the electron transfer efficiency. Ultimately, the fusion of *Mastigocladus laminosus* Fd and LaG-41 Nb results in a functional chimera whose activity is gated by the presence of GFP. This study exemplifies how synthetic biology can achieve systems-level control of biomolecules for real-world applications.

To close, the field of biomolecular technology is undergoing a boom, advancing both fundamental knowledge and opportunities for translational impact. The biomolecules that make up these technologies and the approaches used to engineer them are limitless in scope. Here, we have only scratched the surface, with new advances in biocatalysis, biosensing, and synthetic biology. We hope this collection serves as a launchpad to inspire toolmakers to push the boundaries of innovation and transform how biomolecules are designed and applied across new and unexplored contexts.

